# Unfortunate Accident or Blessing in Disguise? Dramatic Response to Incidental Intrathoracic Delivery of Anti-HER2 Regimen

**DOI:** 10.1200/JGO.2016.008565

**Published:** 2017-03-21

**Authors:** Khurram Tariq, Ryan Keen, Karen Draper, Shou-Ching Tang

**Affiliations:** **Khurram Tariq**, **Ryan Keen**, **Karen Draper**, and **Shou-Ching Tang**, Georgia Regents University Cancer Center, Augusta, GA; and **Shou-Ching Tang**, Tianjin Medical University Cancer Institute and Hospital, Tianjin, China.

Breast cancer is the most frequent form of malignancy in the female population. Despite tremendous advancements in breast cancer screening, metastatic disease remains alarmingly common.^1-4^ In as much as 40% of patients with metastatic breast cancer, pleural involvement may be the initial and only manifestation.^1-4^ Approximately 20% of all patients with breast cancer have either an amplification or an overexpression of the human epidermal growth factor receptor 2 (HER2^+^).^5^ Clinically, the presence of HER2^+^ overexpression translates into a more aggressive disease progression and an overall poor prognosis.^6^ However, the field of oncology has witnessed several exciting developments over the past decade. Clinical trials have shown the superiority of a combined anti-HER2 regimen for both local and advanced breast cancer when compared with alternative therapies.^7-11^ In this case, we present, to the best of our knowledge, the first reported case of accidental intrapleural administration of pertuzumab, trastuzumab, and docetaxel (PTH). Although administered in a nonintentional fashion, we were surprised by the exceptional local control results and the safety of the regimen.

## CASE PRESENTATION

**Fig 1:**
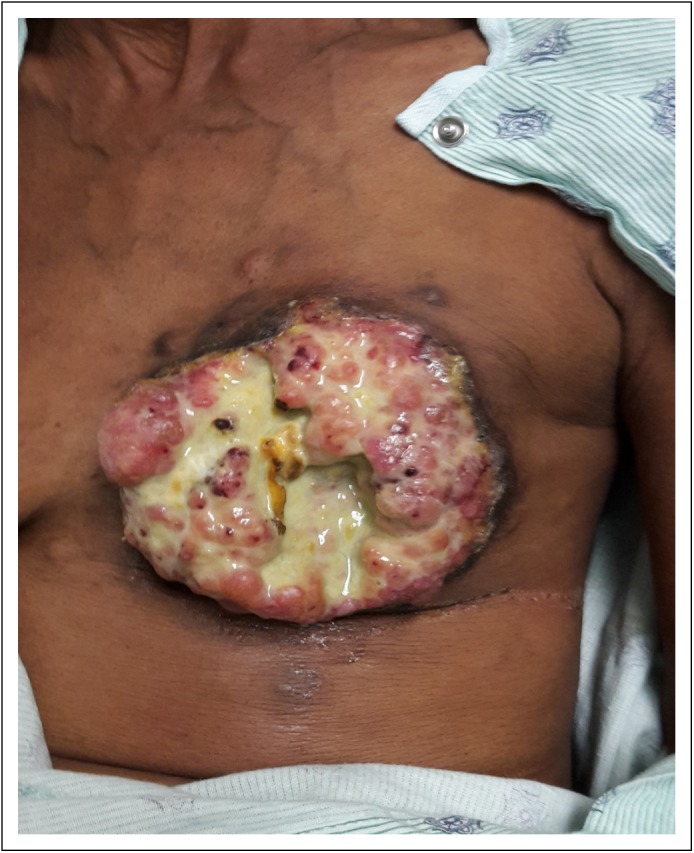
Photo of the patient’s left breast mass on presentation.

**Fig 2:**
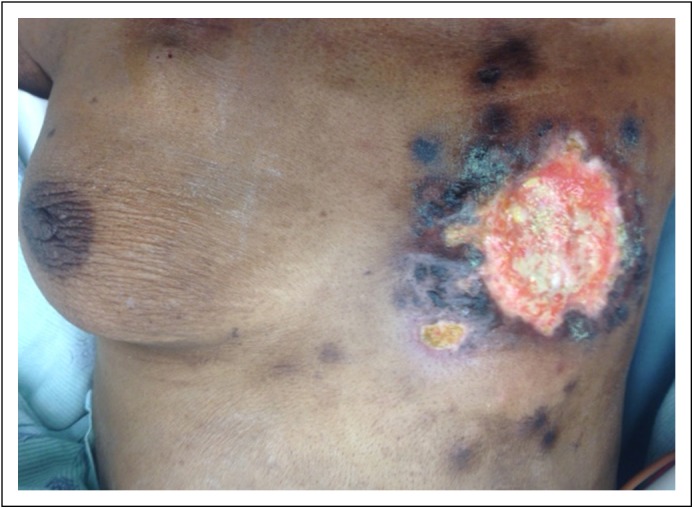
Photo of patient's left breast wound after two cycles of intrapleural pertuzumab, trastuzumab, and docetaxel.

Our patient was a 66-year-old woman who presented with a reported 6-month history of an enlarging ulcerating mass replacing the entire left breast, with drainage of purulent, foul-smelling fluid. A diagnosis of invasive carcinoma of the left breast was confirmed on core biopsy. The tissue sample underwent immunohistochemical testing, which did not reveal the overexpression of estrogen or progesterone receptors. However, the pathologic testing did reveal overexpression of the HER2/neu protein. Physical examination revealed left-sided supraclavicular and bilateral axillary lymphadenopathy, and a positron emission tomography scan revealed widely metastatic disease of the lungs, liver, and bone. The patient received a right internal jugular vein portacath and palliative chemotherapy, which was initiated with PTH 5 days after port insertion. Three weeks after the first chemotherapy administration, there was dramatic regression of the left breast lesion (Figs 1 and 2), as illustrated by pictures taken before and after treatment.

**Fig 3:**
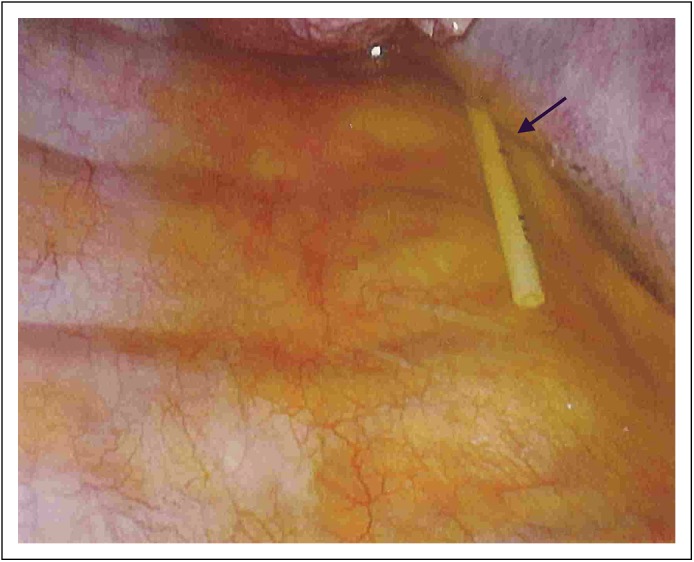
Catheter entering the right pleural cavity posterior to the superior vena cava with pleural effusion fluid.

At the time of administration of the first chemotherapy cycle, blood had been aspirated from the port before infusion. When the patient returned to the chemotherapy infusion center for her scheduled second cycle of PTH, the nursing staff was unable to aspirate blood from the port, but, because it flushed without difficulty, it was used for the second infusion of PTH. The patient once again tolerated the infusion with no unusual symptoms. On her third cycle of chemotherapy, aspiration from the port produced a clear yellow liquid. The patient was sent to the radiology department for assessment of the port. A chest x-ray was obtained, and the position of the port was reported as normal, but a new pleural effusion was noted. A subsequent venogram study showed flushing of intravenous (IV) contrast from the catheter into the right pleural cavity. The intrapleural position of the catheter was confirmed by chest computed tomography scan, and it was apparent that the first two doses of the PTH regimen had been administered directly into the right pleural cavity. The patient was taken to the operating room the next day, and the portacath was removed, with thoracoscopic observation. The catheter was noted to enter the right pleural cavity via a perforation at the junction of the right subclavian vein and the superior vena cava (Fig 3). There was no bleeding from the vein on removal of the portacath, and the pleural fluid was drained. Of note, there were no pleural adhesions observed during the thoracoscopic examination.

The patient was clinically stable after the incident and was able to resume the PTH regimen as scheduled. She was restaged with a computed tomography scan of the thorax, abdomen, and pelvis after the third cycle of PTH, and the results revealed significant improvement in the disease burden of the thoracic cavity; however, disease progression was noted in all other known sites.

## DISCUSSION

Our case is unique because of the intrathoracic administration of the PTH regimen for the treatment of recurrent HER2^+^ breast cancer with large chest wall disease, as well as the distant metastasis. Although we intended to administer the standard IV regimen, the tip of the portacath had been unknowingly placed in the right chest cavity. Consequently, the first two cycles of chemotherapy were administered into the pleural space. Most likely, the blood aspirated from the port before the first dose of chemotherapy was actually blood from perforation of the vein on port insertion within the right chest.

Intrapleural chemotherapy has long been used to treat malignant pleural effusion from various malignancies.^12-14^ To our knowledge, it has never been used for the treatment of chest wall soft tissue malignancy, let alone HER2^+^ metastatic breast cancer. Chest wall soft tissue lesions may have a different biology from the distant metastatic diseases and may respond differently to different chemotherapy dosing and frequency regimens. The pleural cavity may function as a reservoir to deliver a prolonged infusion of low chemotherapy, which is more effective for chest wall soft tissue diseases. Alternatively, intrapleural chemotherapy may provide a better drug bioavailability for chest wall lesions than do agents administered IV. The dramatic improvement in the chest wall lesion in response to the incidental intrapleural PTH in this case seems to support our hypothesis. Indeed, the distant metastatic disease progressed on restaging after the completion of three cycles. It should be noted that docetaxel is considered a vesicant. It causes tissue damage if extravasation occurs during IV administration. It was surprising to note that there were no pleural adhesions after the incidental intrapleural docetaxel infusion and no pain during administration.

Our case strongly suggests the need to further explore the use of intrapleural chemotherapy as a novel treatment modality in metastatic cancer with pleural or chest wall diseases, including PTH for HER2^+^ breast cancer.

In conclusion, advanced HER2^+^ breast cancer is an aggressive disease with an exceptionally poor prognosis if left untreated. Although the use of combination docetaxel with anti-HER2/neu therapy involving pertuzumab and trastuzumab has revolutionized the treatment paradigm, the accidental intrapleural administration of this regimen showed exceptional and rapid local disease response without pleural reaction or adhesion formation. Cavitary chemotherapy in the form of intraperitoneal delivery is being used successfully in the management of metastatic ovarian cancer,^15^ but our case uniquely shows the successful use of intrapleural chemotherapy in the management of metastatic breast cancer. Our case report strongly indicates that further clinical studies of intrathoracic chemotherapy, including the anti-HER2 therapy to control chest wall or intrapleural metastatic cancer, are warranted.
